# From Translation to Treatment

**DOI:** 10.12703/r/12-9

**Published:** 2023-04-28

**Authors:** Xuemin Wang, Christopher G Proud

**Affiliations:** 1 Lifelong Health, South Australian Health & Medical Research Institute, Adelaide SA5000, Australia

**Keywords:** mTORC1, MNK, eIF4E, eIF2B, VWM

## Abstract

Protein synthesis (mRNA translation) plays a crucial role in cell function by shaping the proteome –making all the proteins each cell requires at the right time and in the right quantities and places. Proteins carry out almost every job in the cell. Protein synthesis is also a major component of the cellular economy, using large amounts of metabolic energy and resources, especially amino acids. Accordingly, it is tightly regulated through diverse mechanisms which respond, for example, to nutrients, growth factors, hormones, neurotransmitters and stressful situations.

mRNA translation involves the concerted action of ribosomes, proteins termed translation factors, mRNA-binding proteins and other components. This review will focus on translation factors and their control, and particularly those involved in translation initiation (Figure 1), where ribosomes (beginning with the small 40S subunit) are recruited to mRNAs and then locate the start codon of the coding sequence in a process termed ‘scanning’ (Figure 1A). The elongation and termination phases of translation ensure the correct synthesis of the new polypeptide and its release, along with the ribosomal subunits, from the mRNA when the stop codon of the coding sequence is reached. Translation factors involved in initiation are termed eukaryotic initiation factors (eIFs). Several are subject to control by phosphorylation.

**Figure 1.**
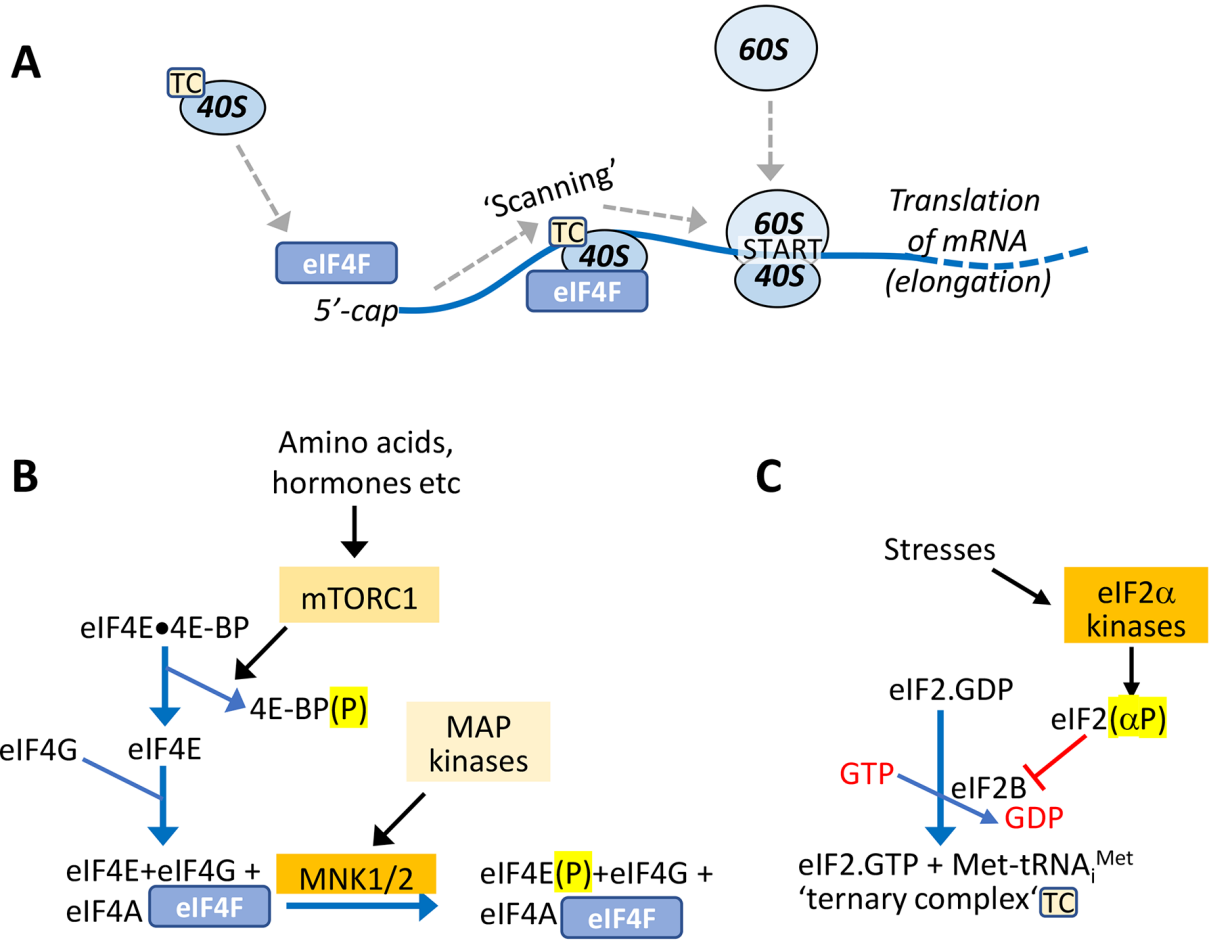
Control of translation initiation. This figure depicts, in a greatly simplified way, the major points about the initiation of mRNA translation and its regulation which are discussed in the text. (**A**) overview of the major steps; TC = ternary complex; note that it is the eIF4E component of eIF4F that interacts with the 5’-cap (see also panel **B**). (**B**) mTORC1 regulates the formation of eIF4F complexes via phosphorylation of 4E-BPs, while MNKs phosphorylate eIF4E. (**C**) eIF2 forms ternary complexes, which require it to be bound to GTP. Regeneration of eIF2•GTP is mediated by eIF2B, and this process is inhibited by phosphorylated eIF2.

This article will focus on the signalling pathways which control mRNA translation. For example, mTORC1 signalling is hyperactivated in many cancers, increasing the availability of eIF4E and promoting translation initiation and the translation of a subset of mRNAs (rather than every mRNA). There is substantial literature describing mechanisms by which translation of individual kinds of mRNAs is controlled, some of which malfunction in human disease.

## Multiple ways to control protein synthesis

Two major stages involved in initiating the translation of any cytoplasmic mRNA are the recruitment of ribosomes and locating the start codon (Figure 1A). Diseases arising, e.g., from mutations in components involved in each of those steps, or in proteins, such as protein kinases, that regulate translation, are known to give rise to diverse human diseases. Our improving knowledge of these steps and their control is enabling us to devise ways to treat or manage those disorders.

Every eukaryotic cytoplasmic mRNA possesses a ‘cap’ structure at its 5’-end, which includes a 7-methylguanosine moiety. eIF4E interacts with the cap and with other eIFs to form the eIF4F complex and enable the recruitment of ribosomes to the mRNA (Figure 1A,B). eIF4E is subject to control in two main ways; through its availability to bind other eIFs, via small phosphoproteins called eIF4E-binding proteins (4E-BPs) and via its own phosphorylation (Figure 1B). 4E-BP phosphorylation is catalysed by a protein kinase complex, the mechanistic target of rapamycin complex 1 (mTORC1). Phosphorylation of 4E-BPs brings about their release from eIF4E making eIF4E available for translation initiation, specifically to bind eIF4G. eIF4G binds an RNA helicase, eIF4A (Figure 1B), to form eIF4F (Figure 1B). Binding of eIF4F to the 5’-end of the mRNA facilitates ribosome recruitment. eIF4A aids this process by ‘unwinding’ any secondary structure in the 5’-UTR, which can inhibit efficient translation of the mRNA.

Another way whereby mTORC1 signalling promotes the translation of a subset of mRNAs is by phosphorylating the protein LARP1 (reviewed 1). When hypophosphorylated, LARP1 binds mRNAs that have a run of pyrimidines at their 5’-end (so-called TOP [= terminal oligopyrimidine] mRNAs). Phosphorylation of LARP1 by mTORC1 induces its release from the cap and TOP sequence, allowing eIF4E to bind and the TOP mRNAs to be efficiently translated. These mRNAs encode ribosomal proteins and some translation factors, allowing mTORC1 signalling to drive ribosome production and thus increase the cell’s capacity for protein synthesis. Indeed, mTORC1 acts as a ‘master regulator’ of cellular function by controlling many aspects of metabolism.

mTORC1 activity depends on an adequate supply of certain essential amino acids and is further stimulated by hormones and growth factors through signalling pathways such as phosphatidylinositide 3-kinase (PI 3-k)/protein kinase B (AKT) or Ras/MAP kinase (ERK). These pathways are frequently activated in cancers due to activating mutations in their upstream components, including receptors, or loss of function of proteins (tumour suppressors) that normally restrain oncogenic signalling. Thus, mTORC1 signalling is hyperactivated in many cancers, increasing the availability of eIF4E and promoting translation initiation and the translation of a subset of mRNAs (rather than every mRNA). There is substantial literature describing mechanisms by which translation of individual kinds of mRNAs is controlled, some of which malfunction in human disease; however, this article will focus on the signalling pathways which control mRNA translation.

## mRNA translation and human disease

Given that mTORC1 signalling controls diverse cellular processes, is its effects on the translational machinery important in cancer? Numerous lines of evidence support this idea. For example, a seminal discovery was that artificial over-expression of eIF4E can transform cells^2^. Indeed, eIF4E is often expressed at high levels in cancer cells. A further striking insight was provided by creating a haploinsufficient eIF4E^+/-^ mouse line^3^. Reducing eIF4E levels by half did not elicit any overt phenotype but did render cells from such mice resistant to oncogenic transformation. Rapamycin, a macrolide compound that interferes with the function of mTORC1, can overcome resistance to anti-cancer drugs in certain models^4^, but its ability to do so is lost when eIF4E is overexpressed. Both these studies indicated that the availability of eIF4E and its control by mTORC1 are central to tumour development.

An interesting approach to damping down eIF4E function is the use of a small molecule (4EGI-1; 5) to block binding of eIF4E to eIF4G. Its use in disease models has generated intriguing data in settings such as neurodegenerative disease^6^, fragile X syndrome^7^ and cancer cells (e.g., 8). In prostate cancer, for example, reducing levels of the androgen receptor increases eIF4E/eIF4G binding and drives faster cell proliferation, effects which are counteracted by 4EGI-1^9^. However, given the likelihood of undesirable effects of impairing this important interaction, such as interference with memory consolidation^10^, it is unclear how applicable this approach would be in disease therapy.

In addition to cancer, mTORC1 signalling, and likely the control of translation, play key roles in other disorders such as cardiac hypertrophy (potentially life-threatening overgrowth of heart muscle,^11^), tuberous sclerosis complex (TSC,^12^), a genetic disorder characterised by benign tumours termed hamartomas), and restenosis after angioplasty, a complication of cardiac surgery^13^.

## Targeting mTORC1 signalling in disease therapy

Several types of mTOR inhibitors have been developed (Table 1). These include rapamycin and its more drug-like analogues (rapalogs), inhibitors of the kinase activity of mTOR and a combined inhibitor, RapaLink1, which contains both rapamycin and a kinase inhibitor coupled by a linker^14^. Therapeutic use of rapamycin and rapalogs has been limited by several factors. These include their ability to (re)activate oncogenic pathways such as PI 3-kinase/AKT and ERK MAP kinase signalling (reviewed 15) and their mechanism of action, which is to occlude access by certain substrates to the activity of mTOR^16^. This means their effects on some mTORC1 effectors, notably 4E-BPs, are weak^17^. Compounds such as BEZ235^18^ (Table 1), which inhibit both PI 3-kinase and mTOR (which are related enzymes), may be of utility in cancer therapy by virtue of their ability both to inhibit two oncogenic pathways and to prevent the consequences of mTOR inhibitor-induced activation of PI 3-kinase/Akt signalling. However, the broad activity of BEZ235 against both PI 3-kinase and mTOR may mean it is poorly tolerated^19^, indicating that more specific agents may be needed to avoid toxicity. Mutations in mTOR can cause rapamycin resistance, a feature which is at least partially overcome by Rapalink1^14^. Interestingly, while mutations that promote PI 3-kinase signalling evoke sensitivity to mTOR inhibitors, simultaneously possessing mutations that activate Ras/Raf signalling overcomes such sensitivity^20^. Thus, genetic analysis of tumours will be valuable in stratifying patients for treatment with inhibitors of mTOR signalling, as is the case for other therapeutic interventions.

**Table 1.** Selected compounds that interfere with the control or function of translation factors. Compounds chosen from among those mentioned in the text, plus compounds with similar or related activities.

Compound	Target	Stage in development	Comments
4EGI-1	Binding of eIF4E to eIF4G	Preclinical	See 5
AZD8055	mTOR	Phase 1 revealed adverse effects	Inhibits kinase activities of mTORC1 and mTORC2
BEZ235	PI 3-kinase and mTOR	Phase 1	Dual inhibitor of these two related kinases
eFT226 (zotatifin)	eIF4A	Phase 2a	Related to Rocaglamide A, a natural product; other natural products also inhibit eIF4A, e.g., hippuristanol, pateamine A
eFT508 (tomivosertib)	MNK1 and MNK2	Phase 2b; other trials underway in oncology	
ISRIB	eIF2B	Preclinical	Promotes eIF2B function
Rapamycin (and related compounds termed rapalogs Temsirolimus Everolimus Ridaforolimus	mTORC1	FDA-approved for restenosis and prevention of kidney graft rejection; >1000 trials underway or completed Temsirolimus has FDA approval for renal cell carcinoma; many further trials underway FDA approved for multiple indications; >100 further trials planned or underway One trial planned	Allosterically inhibit activity of mTORC1 against some substrates (but not all); impairs assembly of mTORC2, and thus signalling through that pathway in the longer term

The roles of mTORC1 in many normal cellular processes lead to undesirable effects, including stomatitis, skin disorders and immunosuppression^21^, which is particularly undesirable in cancer patients. Rapamycin also interferes with the assembly and, thus, the function of mTORC2 in some cells and tissues. Impairment of mTORC2 signalling contributes to its effects, including ‘side-effects’. Rapamycin and rapalogs can impair glycaemic control, raise serum lipid levels and increase triglyceride stores, elicit hepatotoxicity and cause proteinuria^21–23^. At low doses, RapaLink1 is selective for mTORC1, although the dosing window is small^24^.

Despite these problems, rapamycin is in clinical use for restenosis, where it is delivered locally through drug-impregnated stents, prevention of kidney graft immune rejection (a ‘life-or-death’ situation), and some cancers, such as advanced renal cell carcinoma and breast cancer and some kinds of pancreatic cancer^25^. Clinical trials show promise for the management of TSC^26^, although side effects remain an issue. Numerous other clinical trials are under way.

A number of inhibitors of eIF4A have been identified and tested in preclinical cancer models, where they exert anti-tumorigenic effects presumably by altering the spectrum of mRNAs undergoing active translation^27^. This is a promising area for future study; one compound (eFT226; zotatifin, see Table 1) is in clinical trials.

## MNKs, the kinases that phosphorylate eIF4E, are promising drug targets

eIF4E is also regulated by phosphorylation, an event that decreases its affinity for the 5’-cap^28^ (Figure 1B). Phosphorylation of eIF4E is catalysed by the MAP kinase-interacting kinases (MNKs), which as their name suggests, are activated by the (oncogenic) MAP kinase pathway^29^. The precise effect of eIF4E phosphorylation on mRNA translation is still not fully clear, but it appears to promote the translation of some mRNAs, including ones encoding proteins involved in tumour metastasis^30,31^. MNK activity and/or phosphorylation of eIF4E have been shown to be important for tumour initiation and/or progression^32,33^ and metastasis (e.g., 34). Several selective or specific MNK inhibitors have been developed. Use of one of them, eFT508 (tomivosertib, Table 1), has revealed roles for the MNK/eIF4E pathway in the immune checkpoint^35^ and some cancer models (e.g., 36). Interestingly, this MNK inhibitor also showed efficacy in countering neuropathic pain^37^ and relieved deficits, including behavioural ones, associated with Fragile X syndrome^38^.

MNKs are also involved in governing lipid metabolism. While mice fed a high-fat diet (HFD) gain weight and suffer other adverse effects, animals lacking MNKs or mice treated with an MNK inhibitor are protected from HFD-induced weight gain^39–41^. This appears to reflect the control by eIF4E of translation of mRNAs for protein involved in lipid storage and metabolism, such that mice treated with MNK inhibitor show faster oxidative metabolism, likely contributing to their reduced weight gain on an HFD.

Given that MNKs appear to be relatively safe targets for intervention, there is an exciting prospect that MNK inhibitors such as eFT508 (Table 1) may be of value in tackling a range of conditions from malignant disease to metabolic disorders.

## mTORC1 also controls other aspects of protein metabolism

mTORC1 also regulates other components involved in translation, including the elongation process, in that case by controlling the activity of eukaryotic elongation factor 2 kinase (eEF2K)^42^ (Figure 1A). Control of elongation is important in the development of some tumours and underlies sensitivity to mTORC1 inhibitors^43^, but can also help support cell survival in solid tumour cells (reviewed 44), likely by conserving energy and amino acids. Thus, like autophagy, another process controlled by mTORC1, interfering with eEF2K activity appears to represent a ‘double-edged sword’ in cancer therapy.

## The initiator methionyl-tRNA binding step is also a key regulator of protein synthesis

Recognition of the start codon during initiation requires the recruitment to the ribosomes of the specialised initiator tRNA, attached to its cognate amino acid, methionine. Met-tRNA^Met^_i_ is brought to the 40S subunit by eIF2 (a heterotrimer, αβγ), but only when eIF2 is in its active GTP-bound state; the eIF2•GTP• Met-tRNA^Met^_i_ entity is termed a ‘ternary complex’ (TC). The GTP is hydrolysed during initiation so that eIF2 leaves the ribosome as inactive eIF2•GDP. This is then recycled back to active eIF2•GTP by an ancillary factor, eIF2B, a heterodecameric protein (more of which later; reviewed 45; Figure 1C).

This system is regulated in several ways, one being through the phosphorylation of eIF2 on its α-subunit^45^. This phosphorylation event alters the interaction of eIF2 with eIF2B such that it becomes an inhibitor of eIF2B rather than a substrate (Figure 1C). This, therefore, slows down the delivery of Met-tRNA^Met^_i_ to the ribosome and thereby impairs general protein synthesis; however, the translation of a few mRNAs actually *increases* under this condition (by virtue of special features of their 5’-non-coding regions, discussed in 45).

eIF2α can be phosphorylated by any of four different protein kinases, which are regulated in distinct ways^45^ being activated under diverse stressful conditions as part of the ‘integrated stress response’^46^. Their effect is to slow down overall protein synthesis while inducing translation of the kinds of specific mRNAs mentioned above; since some of those mRNAs encode regulators of transcription such as ATF4 and CHOP, this mechanism can turn on the expression of multiple genes to help cells withstand and then recover from the specific stress they are undergoing.

## Diverse disorders result from defective control of eIF2

Mutations in the genes of each of the four eIF2α kinases (termed *EIF2AK1-4)* are now known to lead to diverse human disorders (Table 2;^45^). Some show autosomal dominance, likely because the mutations impair kinase function, and activation of eIF2 kinases requires dimerisation of functionally competent monomers.

**Table 2.** Genetic disorders associated with mutations (etc) in genes linked to eIF2 and eIF2B. Adapted from 45, where relevant citations may be found.

Gene/protein	Type/name of disorder	Clinical features
*EIF2B1*/eIF2Bα	Leukoencephalopathy with vanishing white matter (VWM); also termed childhood ataxia with central nervous system hypo-myelination (CACH)	Phenotype variable in age of onset, severity, and features; sometimes premature ovarian failure
*EIF2B2*/eIF2Bβ		
*EIF2B3*/eIF2Bγ		
*EIF2B4*/eIF2Bδ		
*EIF2B5*/eIF2Bε		
*EIF2S1/*eIF2α	Permanent neonatal diabetes mellitus (PNDM)	Probably permanent diabetes and liver dysfunction
*EIF2S3*/eIF2γ	MEHMO	Mental retardation, epilepsy, hypogonadism/ hypogenitalism, microcephaly and/or obesity
*EIF2AK1*/HRI	-	Delayed motor development, speech and neuromuscular disorders
*EIF2AK2*/PKR	-	Delayed development, leukoencephalopathy, cognitive impairment, plus additional features
*EIF2AK3*/PERK	Wolcott-Rallison	Early onset insulin-dependent diabetes, growth retardation, skeletal dysplasia and neurodegeneration
*EIF2AK4*/GCN2	-	Pulmonary hypertension

The first to be identified is Wolcott-Rallison syndrome which leads to early-onset diabetes, hepatic dysfunction and skeletal abnormalities^47^ and is caused by mutations in the *EIF4A3* gene encoding the endoplasmic reticulum-associated enzyme PERK. There is growing evidence for a role for the involvement of the eIF2α kinases PERK or PKR (which is activated by double-stranded RNA, e.g., during certain viral infections) in neurodegenerative conditions such as Huntington’s or Alzheimer’s Diseases (HD, AD; reviewed 48). Thus, inhibiting them may be of value in managing AD or HD, although given the importance of PERK in secretory cells such as pancreatic β-cells, there are risks in employing PERK inhibitors. A seemingly counter-intuitive approach is to activate PERK^49^; this idea is based on the observation of that partial or complete loss of PERK function is a risk factor for certain neurodegenerative disorders^50^. Initial studies indicate this approach is beneficial in some preclinical models of such disorders^51^. However, effects in other tissues may again limit the applicability of such an approach.

More than twenty years ago, van der Knaap and colleagues^52^ identified that mutations in genes for eIF2B cause a devastating neurological disorder termed leukoencephalopathy with vanishing white matter (VWM, also termed CACH, childhood ataxia with central hypomyelination). Mutations in any of the five subunits of eIF2B can cause VWM (Table 2). There is very substantial diversity in both the phenotype of VWM (in terms of disease onset, course and severity) and the biochemical effects of VWM mutations on the eIF2B protein^53^).

## Inhibiting the integrated stress response

Studies using the compound ISRIB (‘integrated stress response inhibitor’) have provided key insights. ISRIB binds to eIF2B and alleviates the inhibitory effect of eIF2(αP) on eIF2B’s function (reviewed 45). ISRIB enhanced the activity of ‘VWM’ mutant’ eIF2B complexes^54^ and decreased pathological features while improving motor function in a mouse model of VWM^55^.

Certain mutations in the gene for eIF2Bγ (*EIF2S3*) are associated with the disorder MEHMO (microcephaly, epileptic seizures, microcephaly, hypogenitalism, diabetes and obesity;^56^). In an *in vitro* model, features of this disorder were ameliorated by ISRIB^57^. ISRIB has also shown beneficial effects in models of prion disease, amyotrophic lateral sclerosis and Down syndrome (reviewed 45,46). Importantly, a study on prion disease compared the effects of ISRIB and a PERK inhibitor; the latter restored the rate of protein synthesis, but had harmful consequences likely due to effects on secretory tissues such as the pancreas. ISRIB did not fully restore rates of protein synthesis but was neuroprotective while lacking harmful effects on the pancreas^58^.

Furthermore, recent studies have shown that ISRIB can improve cognitive function in aged mice^59^ and in a mouse model of Alzheimer’s Disease^60^. It also benefitted locomotor ability in a murine model of spinal cord injury^61^. While further work is needed to probe the molecular mechanisms involved, it is already clear that ISRIB, or other molecules with similar activity, may be beneficial in treating disorders associated with inappropriate activation of the ISR or mutations in eIF2B/eIF2γ.

Lastly, evidence is emerging that the integrated stress response (ISR) can promote cancer development (e.g., in lung adenocarcinoma driven by mutant *KRAS*^62^), an effect that was countered by ISRIB. A similar situation has been reported for a model of metastatic prostate cancer^63^. Thus, compounds such as ISRIB are of potential value in treating or managing diverse disease states.

In summary, recent advances in understanding the regulation of mRNA translation have both provided important insights into disease mechanisms and pointed the way to potential therapies for these disorders.
